# Long noncoding RNA and mRNA expression profiles following *igf3* knockdown in common carp, *Cyprinus carpio*

**DOI:** 10.1038/sdata.2019.24

**Published:** 2019-02-19

**Authors:** Feibiao Song, Lanmei Wang, Wenbin Zhu, Zaijie Dong

**Affiliations:** 1 Wuxi Fisheries College, Nanjing Agricultural University, Wuxi 214081, Jiangsu, China; 2 Freshwater Fisheries Research Center of Chinese Academy of Fishery Sciences, Key Laboratory of Freshwater Fisheries and Germplasm Resources Utilization, Ministry of Agriculture and Rural Affairs, Wuxi 214081, Jiangsu, China

**Keywords:** RNA sequencing, RNAi, Long non-coding RNAs

## Abstract

As a novel IGF system member, *igf3* plays an important role in gonadal development of teleost fish. Although studies have reported the unusual expression of *igf3* in fish gonad, whether the *igf3* affects the expression of long noncoding RNAs (lncRNAs) in gonad remains unknown. In this study, an *igf3* knockdown common carp (*Cyprinus carpio*) model was established by RNA interference. Then RNA sequencing of *C. carpio* gonad after *igf3* knockdown was performed. A total of 327,169,410 and 306,305,018 clean reads were identified from control and *igf3*-dsRNA interference group, respectively. After a stringent filtering, RNA-seq yielded 14199 lncRNA and 106932 mRNA transcripts with 124 and 353 differentially expressed lncRNAs and mRNAs. Our dataset provides an extensive resource for understanding the potential regulatory molecular mechanism of *igf3* in early stage of gonadal development in *C. carpio*.

## Background & Summary

Insulin-like growth factors (IGFs) are key factors that regulate the growth and reproduction axis. As a new ligand of IGFs, *igf3* plays an important role in the development and maturation of the gonads in fish^1–3^, especially in the maturation of oocytes and the process of spermatogenesis^4,5^. We reported previously that the *igf3* mRNA of common carp (*Cyprinus carpio*) was highly expressed in the gonads and blood, and Igf3 protein was localized in the ovary granulosa cells and testis spermatogonium and spermatids^3^. In addition, *igf3* also serves as a mediator of luteinizing hormone (LH) action in zebrafish (*Danio rerio*) ovulation^6^, and stimulate spermatogonial differentiation by activating β-catenin signaling or through some estrogens^7,8^. To better understand the regulatory pathway of *igf3* in gonadal development of fish, we are interested in the transcriptional regulation of lncRNAs and mRNAs expression profiles following *igf3* knockdown in common carp.

The molecular mechanisms of gonadal development are complex processes that involve in many germ cell-specific gene products in testis or ovary that undergo strict developmental regulations^9,10^. LncRNAs are a novel class of RNAs with the length over 200 bp and without the potential of protein-coding^11^. By now, there are tens of thousands of lncRNAs have been discovered across human, mouse, nematode, zebrafish etc.^12^. For human, 58,648 of genes were classified as lncRNAs^13^. The existing studies show that lncRNAs play a key role in epigenetic, transcriptional and post-transcriptional control of gene expression and maintenance of stem cell, signal transduction, cell proliferation and differentiation, metabolism, and individual development^14–17^. In testis, lncRNAs affect germ cell development and spermatogenesis, such as *tsx, mrh1, dmr and hongES2* ^18–21^. In ovary, many lncRNAs that regulate ovarian development have been identified^22–25^. LncRNA *A-30-P01019163* may affect ovarian cell cycle and proliferation by regulating *p2rx7* expression in the ovary^26^. All of these observations indicate that lncRNAs play important roles in gonadal development.

RNA interference technology is an effective method in studying the gene function of organisms. In this study, we first established a living model of *igf3* knockdown *C. carpio* via double-stranded RNA (dsRNA) injection, and then a high-throughput RNA sequencing (RNA-seq) was employed to profile the primordial gonad transcriptome of *igf3* knockdown and control *C. carpio*. The aims of the experiment were to discover and characterize lncRNAs in carp gonad tissue and identify key genes, lncRNAs, and pathways that are associated with *igf3*. This is the first study to describe the expression profiles of lncRNAs and mRNA in carp gonad with *igf3* knockdown. Our data will provide a very useful resource for studying the functional role of *igf3* in gonadal development.

## Methods

### Animals

The experimental common carp (*Cyprinus carpio*) is a new aquaculture variety derived from the original parents of Jian carp (*C. carpio* var*. jian*), Huanghe carp (*C. carpio haematopterus*) and Heilongjiang common carp (*C. carpio haermatopterus*) after five generations combined selection by FFRC, CAFS. This newly improved strain is designated as FFRC No. 2 strain common carp. It is one of the dominant aquaculture species in China^27^. The *C. carpio* were obtained from the Qiting Pilot Research Station (Yixing, China), which is affiliated with the FFRC. Juvenile fishes at 8 weeks post hatching (10.92 ± 0.14 g) were maintained in the aquaculture experimental facilities during the acclimation and experimental periods under 12-h light/dark cycles at 28 ± 1 °C, and fed twice daily with compound feed (Tech-bank Co., Ltd., Ningbo, China).

### RNA interference (RNAi)

This description of RNA interference is extended from the protocol described in the related research manuscript^28^. The dsRNA of *igf3* was synthesized in vitro using TranscriptAid T7 High Yield Transcription Kit (Thermo Scientific, USA) according to the manufacturer’s instructions. The template for *igf3*-dsRNA synthesis was prepared by amplifying gonad cDNA with the primers *igf3* iF and *igf3* iR (Table 1 (available online only)). The concentration of dsRNA was measured at 260 nm with a BioPhotometer (Eppendorf, Hamburg, Germany), purity and integrity were examined by 1% agarose gel electrophoresis, and then stored at −20 °C until used.

Preliminary experiment showed that the optimum interfering effect was observed at the 24^th^ hour after the intraperitoneal injection of *igf3*-dsRNA with the dose of 5 μg/g body weight. In this experiment, juvenile *C. carpio* were randomly divided into two groups with 15 fishes per group. The experimental group was injected intraperitoneally of *igf3*-dsRNA and the control group was injected intraperitoneally of H_2_O at an equal dose based on body weight. Gonad samples were obtained at 24 h after injection, and 9 fish samples were obtained per group. Gonad samples were snap-frozen in liquid nitrogen and stored at −80 °C until processed. The expression level of *igf3* was tested by qRT-PCR, primers used as shown in Table 1 (available online only).

### Total RNA isolation and qualification

Total RNA was extracted from gonad tissues using TRIZOL (Invitrogen, Carlsbad, CA, USA) according to the manufacturer’s protocol, genomic DNA was removed from RNA sample using DNase I (New England Biolabs). The concentration and integrity of RNA was estimated using the NanoDrop 2000 (Thermo Scientific, USA) and Agilent 2100 Bioanalyzer (Agilent Technologies, CA, USA), respectively. And only high quality samples with an RNA Integrity Number (RIN) value greater than or equal to 6.8 were used to construct the sequencing library.

### Library preparation for lncRNA sequencing

In this study, 3 μg total RNA from each gonad sample was used as the input material for library preparation. Six cDNA sequencing libraries, designated control (control-1, control-2 and control-3) and treatment (*igf3*-dsRNA-1, *igf3*-dsRNA-2, and *igf3*-dsRNA-3), were constructed using NEBNext Ultra Directional RNA Library Prep Kit for Illumina (NEB, Ipswich, MA, USA) after removal of ribosomal RNA by the Epicentre Ribo-zero rRNA Removal Kit (Epicentre, Madison, WI, USA). Then, first strand cDNA was synthesized using random hexamer primers and M-MuLV reverse transcriptase; second strand cDNA synthesis was subsequently performed using DNA polymerase I and RNase H and reaction buffer, dUTP replaced dTTP in the reaction buffer used in second strand cDNA synthesis. The resulting double-stranded cDNA was ligated to adaptors after being end-repaired and A-tailed. Length of 350–400 bp cDNA fragments were preferred and isolated after purifying using the AMPure XP system (Bechman Coulter, Beverly, USA). PCR amplification was performed to enrich cDNA libraries. Finally, PCR products were purified and library quality was assessed on the Agilent Bioanalyzer 2100 system. The libraries were then sequenced on the Illumina HiSeq X platform, generating 150 bp paired-end reads.

### Transcriptome assembly and lncRNA identification

Quality control and reads statistics were determined by FastQC^29^. At the same time, Q20, Q30 and GC contents of the clean reads were calculated, and all the downstream analyses were based on clean reads with high quality. The final clean reads were then mapped to the *C. carpio* reference genome (version 3.0) using Hisat2 aligner. We used reference annotation file of *C. carpio* (version 3.0, provided by CAFS, http://www.fishbrowser.org/database/Commoncarp_genome) to guide the transcripts assembly of each sample with StringTie program (v1.3.3b)^30^. Then the output GTF files were merged into a single unified transcript using stringTie merge function. The merged transcripts were compared to the reference annotation using gffcompare program (v0.10.1, https://ccb.jhu.edu/software/stringtie/gffcompare.shtml). To identify potential lncRNAs, transcripts which were longer than 200 bp and belonged to special class code (“j”, at least one splice junction is shared with a reference transcript; “o”, generic exonic overlap with a reference transcript; “i”, a transfrag falling entirely within a reference intron; “u”, unknown, intergenic transcript; “x”, exonic overlap with reference on the opposite strand) were retained. Furthermore, TransDecoder (version rel16JAN2014) was used to identify transcripts with open-reading frame (ORF), while NONCODE and NR database were conducted to discover known lncRNAs and protein-coding transcript homologs. Additionally, we used Coding Potential Calculator (CPC, version 0.9-r2) to evaluate the coding potential of transcripts. Combined with above computational approaches, the predicted novel lncRNAs had to meet the following requirements: 1)transcripts with an FPKM (fragments per kilobase per million mapped reads) value < 0.1 were removed; 2) transcripts which had ORF or be homologous to known lncRNAs transcripts or protein-coding transcripts were discarded; 3) transcripts with CPC score larger than 0 were abandoned. The remained transcripts from the intersection results were considered as non-coding.

### Differential expression of lncRNA and mRNA

The TPM (Transcripts Per Million) was used to estimate the expression levels of lncRNAs and mRNAs in every sample. Differential expression analyses in the control and treatment groups were performed using the R package (edgeR 3.22.3), an absolute value of |log2 (fold change)| > 1 and an adjusted *P*-value of < 0.05 were set as the filter criteria for significant differential expression. The differential cluster analysis of genes and a volcano plot of differentially expressed lncRNAs and mRNAs were drawn by the E Charts platform (http://www.ehbio.com/ImageGP/index.php/Home/Index/index.html).

### Quantitative real-time-PCR (qRT-PCR) validation

RNA samples from the gonad of 6 individuals used for the RNA-seq were analyzed by qRT-PCR. First-strand cDNA was synthesized using PrimeScript RT Master Mix (Takara, Japan). qRT-PCR was performed on a CFX96 Real-Time PCR Detection System (Bio-Rad, Hercules, CA, USA) using SYBR Premix Ex Taq II (Takara) according to the manufacturer’s protocol. Specific primers of genes and lncRNAs as shown in Table 1 (available online only) were designed using the Primer Premier 5. Each 25 μL reaction volume contained 12.5 μL 2 × SYBR Premix Ex Taq II, 1.0 μL diluted cDNA template (100 ng RNA), 9.5 μL PCR-grade water, and 1.0 μL of each 10 μM primer. The qRT-PCR conditions were as follows: 95 °C for 30 s, followed by 40 cycles of 95 °C for 5 s and 63 °C for 30 s. The relative lncRNA and mRNA expression levels were normalized to *β-actin* and calculated using the 2^−ΔΔCt^ method^31^.

### Statistical analysis

All data are presented as the mean ± standard error of the mean (SEM) and were analyzed using the statistical software SPSS version 22.0 (SPSS Inc., Chicago, IL, USA). Comparisons between the two groups were performed using Student’s *t*-test. Statistical significance was determined at *P* < 0.05.

### Code availability

The code used in this manuscript of R package (edgeR 3.22.3) is available at bioconductor (http://www.bioconductor.org/packages/release/bioc/html/edgeR.html). The gffcompare program (v0.10.1) is available at GffCompare (https://ccb.jhu.edu/software/stringtie/gffcompare.shtml). The TransDecoder (rel16JAN2014) is available at sourceforge (https://sourceforge.net/projects/transdecoder/). The Coding Potential Calculator (0.9-r2) is available at mybiosoftware (http://www.mybiosoftware.com/cpc-0-9r2-assess-protein-coding-potential-transcripts.html).

### Data records

The raw fastq files for the RNA-seq data have been deposited in the NCBI Sequence Read Archive (NCBI-SRA) under accession numbers SRR7944794-SRR7944799 (Data Citation 1), and sample metadata expression estimates can be found on the NCBI Gene Expression Omnibus (Data Citation 2). The information of lncRNAs and mRNAs, information of lncRNA overlap with protein coding genes and annotations of both lncRNA and protein coding genes can be found on the Figshare (Data Citation 3).

## Technical Validation

### Efficiency of *igf3* knockdown in juvenile *C. carpio*

**Figure 1:**
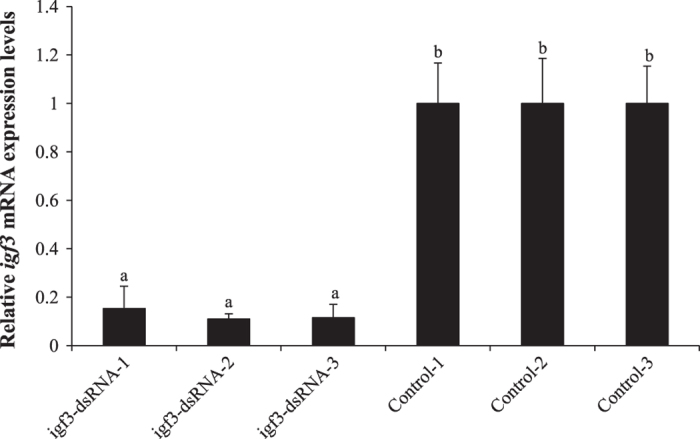
The effect of *igf3*-dsRNA injection analysis by qRT-PCR.

The mRNA expression level of *igf3* was examined by qRT-PCR after RNAi with *igf3*-dsRNA injection. The qRT-PCR results of the *igf3* RNAi were given in Fig. 1, injection of *igf3*-dsRNA resulted in significant down-regulation of *igf3* mRNA expression compared with the control group (*P* < 0.05), and it reached an 87.4% decrease at the 24th hour.

### Overview of RNA-sequencing

In this study, 6 cDNA libraries were constructed using total gonadal RNA from 3 control groups and 3 treatment groups. In total, 663,158,182 raw reads of 150 bp were produced from the 6 cDNA libraries, and 633,474,428 clean reads amounted to 95.02 Gb. More than 80% of the clean reads were mapped to the *C. carpio* reference genome. The average number of sequences that had unique and multiple positions mapped to the reference sequence was 68.17 and 13.41%, respectively. Approximately 34% of the reads substantially mapped to the positive and negative chains of the genome (Table 2).

### Identification of lncRNAs and mRNAs

**Figure 2:**
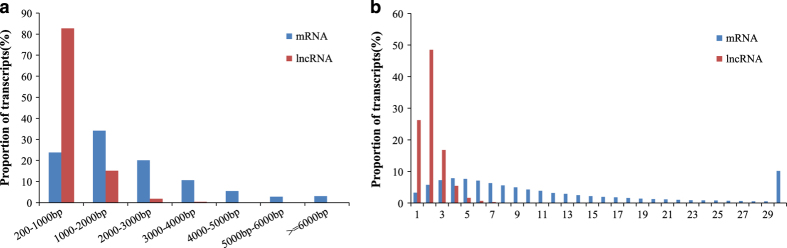
Identification of lncRNAs and mRNAs in *C. carpio* gonad. (**a**) Length of lncRNAs and mRNAs. (**b**) Exonic content of lncRNAs and mRNAs.

RNA-seq yielded 14199 lncRNA and 106932 mRNA transcripts after a stringent filtering process to discard transcripts without the characteristics of lncRNAs and mRNAs. These transcripts were randomly distributed into 50 chromosomes of *C. carpio*, quite notably, the most number of lncRNAs and mRNAs were located in the chromosome 31 and 38 with the percentage of 1.79 and 1.65%, respectively (Information of carp lncRNAs and mRNAs (as surfaced at figshare), Data Citation 3). The length of the lncRNAs ranged from 200 to 5759 bp, with 82.69 and 15.07% of lncRNAs having a length of 200–1000 bp and 1000–2000 bp, respectively, whereas the mRNA transcripts were mainly longer than 1000 bp (Fig. 2a), and the lncRNAs had an average length of 643 bp and 1.6 exons, were less than that of the mRNA (Fig. 2b). In addition, the types of lncRNA overlapping with protein coding genes are ‘j’, ‘o’ and ‘x’ (The information of lncRNA overlap with protein coding genes (as surfaced at figshare), Data Citation 3), and the annotations of both lncRNA and protein coding genes can be found in Data Citation 3 (Annotations of lncRNA and protein coding genes (as surfaced at figshare), Data Citation 3).

### Differential expression of lncRNA and mRNA

**Figure 3:**
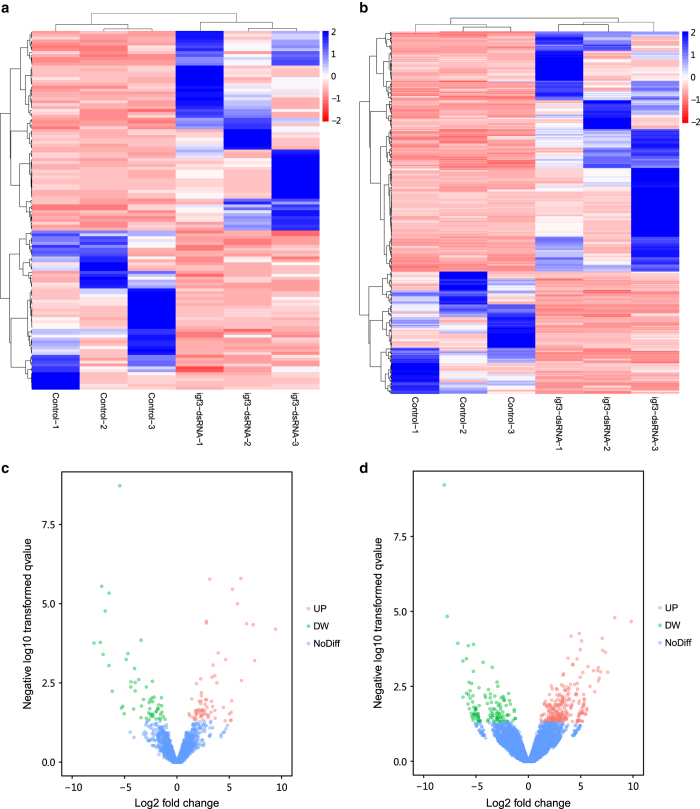
Differentially expressed lncRNAs and mRNAs. (**a** and **b**) are hierarchical clustering of the differentially expressed lncRNAs and mRNAs, blue indicates relative high expression, and red indicates relative low expression. (**c** and **d**) are volcano plots of differentially expressed lncRNAs and mRNAs. X-axis represents fold change (log 2) and Y-axis represents P (−log 10). Red points indicate up-regulated (UP, X-axis > 0) transcripts; green points indicate down-regulated (DW, X-axis < 0) transcripts; blue points indicate no significant difference (NoDiff).

We investigated the differential expression levels of lncRNAs and mRNAs between control group and treatment group. As shown in Fig. 3a,b, hierarchical clustering revealed the significant differential expression (absolute log2 (fold change) > 1, *P* < 0.05) of 124 lncRNAs and 353 mRNAs. In differentially expressed lncRNAs, 69 lncRNAs were up-regulated, whereas 55 lncRNAs were down-regulated in the treatment group (Fig. 3c). Furthermore, among the differentially expressed mRNAs, 234 mRNAs were up-regulated, whereas 119 mRNAs were down-regulated (Fig. 3d). Among these differently expressed genes, there were some key genes associated with gonadal development, such as *fibroblast growth factor receptor 2* (*FGFR2*), *fibronectin-like*, *serine/threonine-protein phosphatase 4*, *serine-rich adhesin*.

**Figure 4:**
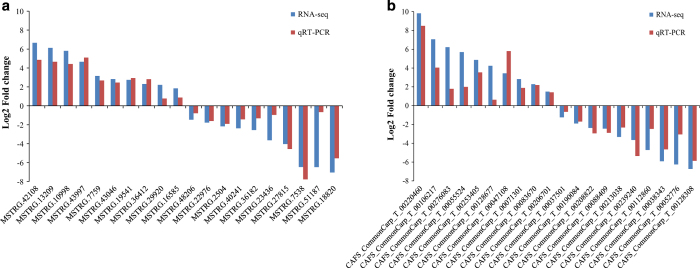
Illustrating of qRT-PCR confirmation for RNA-seq. Validation of 40 selected differentially expressed lncRNAs (**a**) and mRNAs (**b**). The lncRNAs and mRNAs were expressed as log2 (fold change) values (RNA-seq) and −ΔΔCt values (qRT-PCR).

To further evaluate the reliability of RNA sequencing, 20 differentially expressed lncRNAs (10 up-regulated and 10 down-regulated) and 20 differentially expressed mRNAs (10 up-regulated and 10 down-regulated) were randomly selected to validate the relative expression levels in the gonad of the control and treatment group using qRT-PCR. As shown in Fig 4, the trend of differential expression of these 40 randomly selected genes (lncRNAs, Fig. 4a, mRNAs, Fig. 4b) is 100% consistent with our RNA-seq data, indicating that the RNA-seq data was reliable.

## Additional Information

**How to cite this article**: Song, F. *et al*. Long noncoding RNA and mRNA expression profiles following *igf3* knockdown in common carp, *Cyprinus carpio*. *Sci. Data*. 6:190024 https://doi.org/10.1038/sdata.2019.24 (2019).

**Publisher’s note**: Springer Nature remains neutral with regard to jurisdictional claims in published maps and institutional affiliations.

## Supplementary Material



## Figures and Tables

**Table 1:** Primer sequences used in this study.

Gene ID			Primer sequnces (5' → 3')	Product size	Application
igf3	igf3 iF	F	TAATACGACTCACTATAGGGTGTACTGCGTCCTGATCCTG	482 bp	RNA interference
	igf3 iR	R	TAATACGACTCACTATAGGGGAAGGTTGCTGCTGTGTTGA		
	igf3	F	AGCAGCGATACCAGAAGCAT	91 bp	qRT-PCR
	igf3	R	GAGTTCCACCGGTAAAGCGT		
lncRNA	MSTRG.42108	F	GCTCCAGCTTGATCCTCATC	81 bp	
		R	GAGACGAGCTGAAGCCTTTC		
	MSTRG.13209	F	CATTCTCCATGTAGGGCTCC	143 bp	
		R	ACTGGAGGAGCTGAGAACCA		
	MSTRG.10998	F	ACCTTTAAACAAACGGCACG	150 bp	
		R	CCAACTGGGATCCTGAAGAA		
	MSTRG.43997	F	GACACCTTGGGAGGAACTGA	198 bp	
		R	CTTTGCTTTTCACCTCAGGG		
	MSTRG.7759	F	GCAAGCCATCTCTCGACTCT	95 bp	
		R	AGCAGCATCTCCTTCAGCTC		
	MSTRG.43046	F	AGGAGTGAAGTCCTGAGGCA	148 bp	
		R	GATCCCCGTTCACTGTCTGT		
	MSTRG.19541	F	AGCCGACAATAGAGCGGACT	166 bp	
		R	CAGAATCGCTCCCGTTTGAAG		
	MSTRG.36412	F	AAGGCCACGAGATGAAGAGA	127 bp	
		R	GCCCAGCAATAAAAGGAACA		
	MSTRG.29920	F	AAGGGGGAGGTCAACTTGGT	103 bp	
		R	ACAGAGCCTGGAGAGCCATT		
	MSTRG.16585	F	GTCTTAGGAGGACGGGACGA	125 bp	
		R	CCGTGTCACGTGAGTTCTCC		
	MSTRG.48206	F	GCCCCTTGTGTTTTTCTCAA	169 bp	
		R	TCATTTTGGAGGGTGGAAAT		
	MSTRG.22976	F	GCTCCAGTGGACCGTAAAGA	92 bp	
		R	CAGATGGAGGTCCTGGAGAA		
	MSTRG.2504	F	ACAAGCTTTGGTCTGGACATTT	99 bp	
		R	GCTCTTCTGTTCATTTCGACCT		
	MSTRG.40241	F	AACGCCATCTTCATTCGTGT	144 bp	
		R	AAAACTCCTGCTGAAGCGAC		
	MSTRG.36182	F	GCGAAGAAGAACAGTTTGCC	155 bp	
		R	ACAATCCTTCCCGAGTCAGA		
	MSTRG.23436	F	TTTTGTAACCCCCAGCTTTG	128 bp	
		R	TTTACAGACCCCCTTTCCCT		
	MSTRG.27815	F	TCAGGGGAAGTGTTGCTTCT	95 bp	
		R	GCTGAGCCGTTTCAGAAAGT		
	MSTRG.7538	F	AATGGCTGGGTGTGTGTGTA	181 bp	
		R	TGTCGAATATCTGCGGGAAT		
	MSTRG.51187	F	AGACGGGGGCGAGAATACAA	120 bp	
		R	CACTAATCCTGGCCCTGGGA		
	MSTRG.18820	F	GGAGACAGCAGGGAACACAT	178 bp	
		R	GTCCGTGTTTTGCTCCTCAT		
mRNA	CAFS_CommonCarp_T_00220460	F	CCCGTGTGTTTCATTGCTGA	200 bp	
		R	GTGCAGGCAGGATCATTGAG		
	CAFS_CommonCarp_T_00106217	F	TGCTATCGAGTGGCAAACTG	221 bp	
		R	TTAAGGGGAGGAGAGCCATT		
	CAFS_CommonCarp_T_00276083	F	CTGGACCAAGAGTGGTGGTT	137 bp	
		R	CACCAAACTCCCTCCTGAAA		
	CAFS_CommonCarp_T_00055524	F	CTGGGAGGTGGACAAGTCAT	228 bp	
		R	TTCTGGAGGTGGCGTTAGTT		
	CAFS_CommonCarp_T_00253405	F	ACACCCCACCTCACAGAGAC	143 bp	
		R	GGTCACCAACCTGTTCTCGT		
	CAFS_CommonCarp_T_00128677	F	AAACCTACACGTGTGGAGCC	194 bp	
		R	CGGTACGTGGTTCCTCTGTT		
	CAFS_CommonCarp_T_00047108	F	ATTCGGGCGTAGCACAATAC	81 bp	
		R	ATGTCACTTTCTGGGATCGG		
	CAFS_CommonCarp_T_00071301	F	CTCAGGGGGAAAAACAAACA	123 bp	
		R	AAACTCCGTGCAATACGACC		
	CAFS_CommonCarp_T_00083670	F	AGACGAAGGAGGGAGGAGAG	179 bp	
		R	CCGAGGATGATCTCACCAGT		
	CAFS_CommonCarp_T_00206701	F	CTATGACAAAGCCTTCCCCA	131 bp	
		R	GCTGACGTTCTTTTTCGAGG		
	CAFS_CommonCarp_T_00037501	F	TGCATCAGAGTTTCACTGCC	117 bp	
		R	GACGGACTGACAGCAGAACA		
	CAFS_CommonCarp_T_00190084	F	CACCGGTCAGAAACCGTACT	165 bp	
		R	ATCCGCTTGTGGATTTTCAG		
	CAFS_CommonCarp_T_00208822	F	GCAAAAGAGTGTGTGCAGGA	166 bp	
		R	AGAGTTTAAGCGGCTCCACA		
	CAFS_CommonCarp_T_00088409	F	CAGCATTCTGAAAGGAAGGC	167 bp	
		R	ATCCTGTTTTCGCCAACATC		
	CAFS_CommonCarp_T_00213038	F	GAGACCCAAAATCAAGCCAA	125 bp	
		R	TACGGTGTGGTGTTGAGCAT		
	CAFS_CommonCarp_T_00239240	F	CTGAAGCAGGAGTACCAGCC	87 bp	
		R	GTCAGCACCTTTACCCAGGA		
	CAFS_CommonCarp_T_00112860	F	GCCAAGCACAACACTGCTTA	152 bp	
		R	GGACAACTTGGGGAGTTTGA		
	CAFS_CommonCarp_T_00038343	F	TCAGAACGTGGTGTCTGAGC	181 bp	
		R	TTGTACGAGGAGCTGCAATG		
	CAFS_CommonCarp_T_00052776	F	CGAAAACCCAGCAGTTCTGT	149 bp	
		R	TGCTGCCCTCTCGATAACTT		
	CAFS_CommonCarp_T_00128308	F	GTGATCAGCGCTGTGATGTT	122 bp	
		R	CCTGTCACGAGAATGCAGAA		
β-actin	β-actin	F	GCTATGTGGCTCTTGACTTCGA	89 bp	
		R	CCGTCAGGCAGCTCATAGCT		

**Table 2:** Alignment of statistical results of reads.

Sample name	Control-1	Control-2	Control-3	igf3-dsRNA-1	igf3-dsRNA-2	igf3-dsRNA-3
Raw reads	103838052	129702320	109996624	98107722	130263708	91249756
Clean reads	99410868	123563848	104194694	94321406	122675946	89307666
Clean base (G)	14.91	18.53	15.63	14.15	18.4	13.4
Total mapped reads	81530347(82.01%)	99404340(80.45%)	85870625(82.41%)	77049685(81.69%)	99493319(81.10%)	73064096(81.81%)
Multiple mapped	12853963(12.93%)	18112363(14.66%)	12915566(12.40%)	15084557(15.99%)	15762693(12.85%)	10390489(11.63%)
Uniquely mapped	68676384(69.08%)	81291977(65.79%)	72955059(70.02%)	61965128(65.70%)	83730626(68.25%)	62673607(70.18%)
Read-1 mapped	34516969(34.72%)	40477251(32.76%)	36673789(35.20%)	31095154(32.97%)	41919370(34.17%)	31496714(35.27%)
Read-2 mapped	34159415(34.36%)	40814726(33.03%)	36281270(34.82%)	30869974(32.73%)	41811256(34.08%)	31176893(34.91%)
Reads map to ‵+′	34383536(34.59%)	40550020(32.82%)	36580679(35.11%)	31056772(32.93%)	41822537(34.09%)	31350486(35.10%)
Reads map to ‵−′	34292848(34.50%)	40741957(32.97%)	36374380(34.91%)	30908356(32.77%)	41908089(34.16%)	31323121(35.07%)
Notes: Raw reads: The number of total reads. Clean reads: The number of clean reads. Clean base (G): The data of clean reads. Total mapped reads: The number of sequences matched to the genome. Multiple mapped reads: The number of sequences that had multiple positions mapped to the reference sequence. Uniquely mapped reads: The number of sequences that had unique positions mapped to the reference sequence. Read-1, read-2 mapped: The number of read-1 and read-2 that locate on the genome; the statistical proportion of the two parts should be substantially the same. Reads map to '+': The number of reads mapped to the positive strand of the genome. Reads map to '-': The number of reads mapped to the negative strand of the genome.						
